# Improving the foundations of bibliometric analysis: a call for search strategy standardization

**DOI:** 10.1097/JS9.0000000000002991

**Published:** 2025-07-08

**Authors:** Nan Zhang, Haiyang Wu, Cheng Li

**Affiliations:** a Department of Gynecological Tumor Ward, The Third Affiliated Hospital of Zhengzhou University; b Department of Orthopaedics, The First Affiliated Hospital of Zhengzhou University, Zhengzhou, China; c Department of Spine Surgery, Wangjing Hospital, China Academy of Chinese Medical Sciences, Beijing, China; d Center for Musculoskeletal Surgery (CMSC), Charité-Universitätsmedizin Berlin, Corporate Member of Freie Universität Berlin, Humboldt University of Berlin, Berlin Institute of Health, Berlin, Germany

*Dear Editor*,

We read with great interest on the publication by Zhang et al^[1]^ titled “Global research trend of graphene application in breast cancer: a bibliometric analysis,” which has published in the issue of *International Journal of Surgery*. This bibliometric analysis offers a comprehensive overview of graphene’s current applications and research trends in breast cancer, while also highlighting future prospects. Undoubtedly, the results provide key guidance for its utilization and potential advancements in this field. However, we have different suggestions for search terms and search queries for this study.

The validity and reliability of any bibliometric analysis are fundamentally contingent on the methodological rigor underpinning the study, with the data retrieval phase constituting its foundational pillar. The construction of a comprehensive and representative corpus of literature is a prerequisite for all subsequent analytical procedures, including co-occurrence analysis, co-citation mapping, and clustering^[2]^. A meticulously crafted search strategy ensures the exhaustive capture of literature pertinent to the research topic, whereas a flawed retrieval protocol may introduce systematic bias, potentially leading to distorted conclusions and compromising the scientific integrity of the study. Consequently, the formulation of the search strategy, particularly the selection and logical combination of search terms, represents one of the most critical and intellectually demanding components of bibliometric research.

The selection of keywords and the construction of search queries lie at the heart of any bibliometric retrieval strategy^[3]^. Overly broad search terms may introduce a substantial volume of irrelevant literature, commonly referred to as “noise,” whereas overly restrictive terms risk omitting seminal or thematically pertinent studies. In the biomedical domain, the terminological landscape is notably complex and highly specific, with a single concept often represented by multiple synonymous or closely related expressions. To ensure comprehensive coverage, it is essential to include all relevant lexical variants, thereby minimizing the likelihood of missing pertinent literature due to terminological limitations. In the present study, the authors employed the search formula “(breast OR mammary) AND (cancer OR tumor OR carcinoma OR neoplasm OR tumorous OR neoplastic)” to identify literature related to breast cancer. However, this formulation may not fully capture the breadth of relevant research and is likely to exclude numerous potentially significant publications. To enhance retrieval efficacy, additional terms such as “anticancer,” “tumour,” “neoplasm,” and “oncology” should be incorporated into the search syntax. Furthermore, given the frequent alternation between singular and plural forms in scientific writing, the use of wildcards “*” is recommended. For instance, the term “tumor*” would retrieve both “tumor” and “tumors.” Accordingly, we propose the following optimized search strategy for breast cancer: (breast* OR mammary*) AND (anticancer* OR cancer* OR oncology* OR tumor* OR tumour* OR neoplasm* OR neoplastic*).

The choice of search fields exerts a profound influence on the accuracy and relevance of retrieved results. Many researchers conducting searches within the Web of Science (WoS) database tend to rely on the “Topic” (TS) field^[4,5]^. However, the “Topic” field encompasses a broad array of sources, including the title, abstract, author keywords, and the algorithmically generated “Keywords Plus.” Notably, “Keywords Plus” are not supplied by the original authors, and their algorithmic derivation lacks transparency. As a result, they frequently introduce literature of limited thematic relevance, thereby contaminating the dataset and undermining the precision of subsequent analyses. Such a practice runs counter to the foundational principle of scientific research, that of exerting strict control over data provenance. This methodological oversight partly explains why the search strategy in the present study retrieved as many as 1395 publications ostensibly related to breast cancer. In reality, research involving graphene in the context of breast cancer is far less prevalent. In contrast, restricting the search to more focused fields, such as “Title” and “Abstract,” or even exclusively the “Title” field, yields a more targeted and thematically coherent dataset, thereby enhancing the robustness and specificity of bibliometric insights.

As a powerful tool for research evaluation and knowledge discovery, bibliometrics is intrinsically dependent on the quality of its underlying data sources. The validity and scientific credibility of bibliometric analyses hinge upon the implementation of accurate, comprehensive, and reproducible search strategies. There is a pressing need within the academic community for the establishment of standardized reporting guidelines for bibliometric studies, akin to the PRISMA statement for systematic reviews^[6]^. Such guidelines should mandate the transparent and detailed disclosure of key methodological elements, including the choice of databases, complete search queries, search dates, and inclusion and exclusion criteria, to enhance the transparency, reproducibility, and interpretability of bibliometric research. Beyond traditional keyword-based retrieval, future studies should explore more advanced information retrieval techniques. These include early probabilistic models, latent semantic analysis that leverages semantic structures, and emerging retrieval-augmented generation methods powered by machine learning and large language models. While such technologies offer the potential to overcome challenges related to synonymy and polysemy, and to achieve more intelligent and precise literature identification, they also introduce new sources of bias and methodological complexity. Moreover, bibliometric researchers are encouraged to collaborate closely with domain experts and information specialists. Domain experts can provide essential conceptual and terminological insights, while information specialists bring expertise in constructing robust and efficient search strategies tailored to complex research questions.

In sum, the current landscape of bibliometric research is marked by widespread inconsistencies in the reporting and implementation of search strategies, which significantly undermines the interpretive value of many studies. We call on researchers, journal editors, and peer reviewers to collectively elevate methodological standards in bibliometric inquiry and to support the development and adoption of standardized reporting practices. Only through such concerted efforts can bibliometrics fulfill its promise as a rigorous scientific discipline, one capable of illuminating the structure of knowledge with clarity and precision, and of informing research policy and future scholarly directions in a meaningful way. Transparency In The reporting of Artificial Intelligence – the TITAN guideline^[7]^.

## Data Availability

The datasets generated and/or analyzed during the current study are available from the corresponding author by reasonable request.
